# Numerical Investigation into the Development Performance of Gas Hydrate by Depressurization Based on Heat Transfer and Entropy Generation Analyses

**DOI:** 10.3390/e22111212

**Published:** 2020-10-26

**Authors:** Bo Li, Wen-Na Wei, Qing-Cui Wan, Kang Peng, Ling-Ling Chen

**Affiliations:** 1 State Key Laboratory of Coal Mine Disaster Dynamics and Control, School of Resources and Safety Engineering, Chongqing University, Chongqing 400044, China; libo86@cqu.edu.cn (B.L.); 201920131062@cqu.edu.cn (W.-N.W.); e0501183@u.nus.edu (Q.-C.W.); 20182013004t@cqu.edu.cn (L.-L.C.); 2 School of Resources and Safety Engineering, Central South University, Changsha 410083, China

**Keywords:** hydrate, depressurization, entropy generation, heat transfer, energy loss

## Abstract

The purpose of this study is to analyze the dynamic properties of gas hydrate development from a large hydrate simulator through numerical simulation. A mathematical model of heat transfer and entropy production of methane hydrate dissociation by depressurization has been established, and the change behaviors of various heat flows and entropy generations have been evaluated. Simulation results show that most of the heat supplied from outside is assimilated by methane hydrate. The energy loss caused by the fluid production is insignificant in comparison to the heat assimilation of the hydrate reservoir. The entropy generation of gas hydrate can be considered as the entropy flow from the ambient environment to the hydrate particles, and it is favorable from the perspective of efficient hydrate exploitation. On the contrary, the undesirable entropy generations of water, gas and quartz sand are induced by the irreversible heat conduction and thermal convection under notable temperature gradient in the deposit. Although lower production pressure will lead to larger entropy production of the whole system, the irreversible energy loss is always extremely limited when compared with the amount of thermal energy utilized by methane hydrate. The production pressure should be set as low as possible for the purpose of enhancing exploitation efficiency, as the entropy production rate is not sensitive to the energy recovery rate under depressurization.

## 1. Introduction

Natural gas hydrate (NGH) is a type of solid crystalline materials composed of water and small-molecule gases (such as CH_4_, C_2_H_6_, and CO_2_) under appropriate thermodynamic conditions of high pressure and low temperature [1]. The majority of naturally occurred NGH is methane hydrate, and two natural circumstances are favorable for the maintenance of its stability: the deep ocean floors and the permafrost areas [2], where a huge amount of methane gas (more than 10^15^ m^3^) has been verified to be trapped in methane hydrates [3,4]. Such enormous energy reserves have attracted worldwide attentions for the development of NGH as a new and promising energy source to satisfy the ever-increasing energy requirement of our human beings [5]. As gas molecules can be selectively encapsulated in the hydrogen-bonded cages of water [6], a variety of innovative techniques have been introduced for application in many industrial areas based on hydrate crystallization, such as methane separation from mixed gas [7,8], greenhouse gas capture [9,10], desalination of seawater [11,12], and storage and transportation of natural gas [13,14].

For the sake of efficient energy recovery from gas hydrates, several solutions have been suggested based on the principle of destructing the stability situations of NGH systems for fluid extraction [15], including the depressurization [16,17,18,19], the thermal stimulation [20,21,22], the inhibitor injection [23,24,25], and the gas exchange method [26,27]. Due to the technical simplicity and the low external energy demand of the depressurization method, it is widely recognized as the simplest and most promising strategy for hydrate exploitation [28,29]. The enthalpy of hydrate decomposition under depressurization is primarily originated from two aspects: the continuous heat supply from the external environment and the sensible heat of the hydrate deposit [16,30]. The gas extraction rate is usually restricted by the limited thermal conductivity of the hydrate-bearing sediments [31]. In actual mining, hydrates are relatively dispersed and low in content. Pure depressurization-induced mining is not suitable for the development of low-saturation hydrate deposits [32]. Moreover, the issues of hydrate reformation [33] and ice involvement [34,35] due to inadequate heat supply may be the main obstacles hindering sustainable hydrate dissociation by depressurization. Such undesirable phenomena could be suitably avoided by optimizing the operation procedures during the depressurization process [36].

Due to the endothermic properties of NGH dissociation, how the dissociation process is dominated by the heat transfer behaviors in hydrate deposits is a critical and fundamental issue [1]. Zhao et al. [37] implemented a sequence of simulations to figure out the influences of heat transfer on gas hydrate decomposition. It was found that a part of the dissociation heat was provided by the hydrate deposit in the form of sensible heat decline, and this process was affected by the content of water in the pores and the composition of the porous media. Wan et al. [15,31] quantitatively calculated various heat flows during hydrate mining under different conditions of depressurizing and wellbore heating in a high-pressure vessel. The heat conducted from the constant-temperature boundary was found to promote the dissociation in the early stage, and it would be weakened quickly once the injected heat played a more dominating role. According to the analyses of six hydrate exploitation experiments with a variety of methods and wellbore layouts, Liu et al. [38] concluded that it was especially important to take advantage of the heat provided by the outside environment as much as possible for raising the energy efficiency. Tupsakhare et al. [39] discovered that the transportation of the liquid water could carry more heat to distant areas through convection and thus enhance the gas recovery. By conducting numerical analyses of the dissociation properties of gas hydrate around ice point, Li et al. [29] arrived at a conclusion that the released latent heat during ice generation could promote the dissociation rate to some extent when the blockage effect of ice was not pronounced. Similar phenomena were also noticed by Wang et al. [40] when the gas hydrate was decomposed below quadruple point. Konno et al. [41] used HiGUMA, the world’s largest reservoir simulation container, for gas hydrate dissociation analysis by one-step depressurization, multi-step depressurization and depressurization below the quadruple point. The results showed that the latent heat of ice can be effectively used for hydrate dissociation.

As another important parameter of thermodynamics, entropy generation is an effective index of the irreversibility of various energy conversion processes. The analysis of entropy generation is a useful solution for identifying the major sources of irreversibilities [42], which can enlighten us on how to optimize the operating conditions and thus significantly improve the energy recovery performance. Entropy generation is an indicator of the loss of thermodynamic usefulness, and higher entropy generation indicates larger amount of irreversible energy consumption [43]. Assessment of the entropy generation during hydrate exploitation could help to reduce the irreversible energy loss and thus optimize the extraction process of gas hydrate. However, all of the studies reviewed above are mainly based on the principle of the first law of thermodynamics (i.e., the law of energy conservation). Little attention has been paid to the irreversible properties (characterized by the second law of thermodynamics) during gas production from hydrate dissociation due to absence of perfect theory of entropy production in this area. The above studies indicate that heat transfer is closely related to the decomposition of hydrate. The study of heat transfer can provide guidance for efficient hydrate development.

Recently, Feng et al. [43,44] proposed a mathematical model for calculating the entropy production during gas hydrate exploitation in two different large-scale hydrate simulators with configurations of horizontal well. As an approximate approach, the reactors were divided into several tiny parts according to the distribution of thermocouples, and the total entropy production was determined by adding the entropy generation of each separate part. However, due to the limited number of thermocouples in the reactor, such treatment may not be accurate enough because the distributions of the pressure and temperature as well as various materials in the pores are very possible to be non-uniform in each single unit. It would be more rigorous and precise to establish a robust and universal entropy generation model and then combine it with numerical simulation to obtain the real-time entropy production properties during gas extraction from hydrate dissociation. To date, such an aspect has been rarely involved in the literature for the performance evaluation of gas hydrate exploitation. Furthermore, the relevance of entropy generation to heat transfer also remains unclarified in the area of gas hydrate development. It can be concluded from the above studies that heat transfer is a key factor in hydrate decomposition, and entropy generation analysis would be an effective method for optimizing the extraction process of gas hydrate, especially when it is combined with numerical simulation.

Thus, this study aims to carry out numerical analysis of the heat transfer and entropy generation characteristics of methane hydrate dissociation by depressurization in a pilot-scale hydrate simulator (PHS) based on the experimental results reported by Li et al. [16]. A mathematical model specifically used for calculating the heat transfer and entropy generation is established, and then it is combined with the Tough + Hydrate code [45] for the first time to conduct the numerical simulation. The simulated results including the gas recovery volume and the dissociated hydrate mass are compared with the experimental and numerical results reported by Li et al. [16]. Subsequently, the dependence of hydrate dissociation on the heat conduction of the boundary and the sensible heat variation of the system is analyzed. The evolutions of the entropy production of the methane hydrate, the surrounding environment, the water and gas, the sandy sediments, and the whole system are obtained during hydrate exploitation under varying production pressure, and their dependences on heat transfer are investigated. Moreover, detailed discussion has also been performed about the relationships among the energy recovery rate, the entropy production rate, and the dissociation driving force.

## 2. Experimental and Numerical Simulations

### 2.1. Experimental Device and Dissociation Results

**Figure 1:**
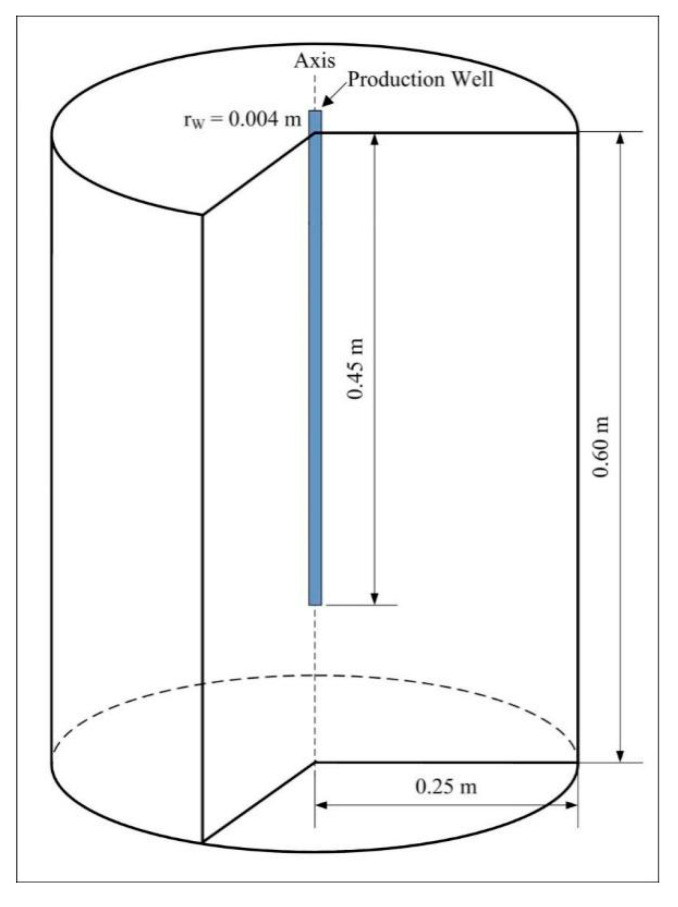
Schematic of the pilot-scale hydrate simulator and the vertical well.

The internal geometric structure of the pilot-scale hydrate simulator (PHS) employed for the hydrate formation and decomposition experiments of Li et al. [16] is displayed in Figure 1, and the full description with respect to this experimental device can be found in the same literature. The core component of this system is a cylindrical stainless vessel which can undergo a maximum pressure of 30 MPa. The valid radius and height of the inner reactor are 0.25 m and 0.60 m, respectively, and the available volume of the container is 117.8 L. A vertical well, of which the length is 0.45 m and the radius is 0.004 m, is placed at the axis of the vessel for fluid production. The whole reactor is encompassed by a cooling device which provides the constant temperature conditions for hydrate dissociation. A total of three experimental cases of hydrate exploitation by depressurization have been conducted in the PHS by Li et al., and the kinetic properties of hydrate dissociation have also been investigated through numerical simulation using the Tough + Hydrate code [16]. However, they did not pay attention to the heat transfer and entropy production behaviors during gas recovery from this large hydrate deposit.

According to the experimental procedure implemented by Li et al. [16], the course of hydrate decomposition is composed of two stages: the residual gas and mixed gas extraction stage, and the stable depressurization stage. Considering that the duration of the first period is much shorter than the second one, we are only focused on the analysis of the hydrate exploitation behaviors in the stable depressurization stage, during which the wellbore pressure is kept invariable. Table 1 provides a summary of the experimental results of gas hydrate development in this stage in the PHS. Δ*t* is the duration of stable depressurization in each experimental case. *P*_0_ and *T*_0_ stand for the initial pressure and temperature of the hydrate deposit, respectively. *P_Well_* is the production pressure of the wellbore, and *T_eq_* represents the gas hydrate equilibrium temperature at the corresponding *P_Well_*. The initial phase saturations of hydrate, gas and water are represented by *S_H_*_0_, *S_G_*_0_ and *S_W_*_0_, respectively. *T_B_* is the environment temperature, and it is fixed at 7.00 °C in all the three runs. *V_P_* is the accumulative volume of the recovered methane in this stage.

### 2.2. Numerical Code and Kinetic Model

The numerical analysis implemented in this study is achieved by employing and updating the parallel edition of the Tough + Hydrate (T + H) code [45]. This code is particularly tailored to the requirements of modeling the phase transformation features of methane hydrate in intricate geological environment. The interdependent physical and chemical processes of heat and four substances, including hydrate, methane, water, and chemical inhibitors, can be described by this code in four kinds of states (i.e., aqueous liquid, gas, hydrate, and ice). Confidence in the adoption of this code for the modeling and forecasting of methane hydrate phase transition properties grows with successful experimental validations in various laboratory-scale hydrate deposits [15,16,22,28]. However, the irreversibilities during hydrate development have not been considered in this code due to the absence of a reliable entropy production model.

The phase transition kinetics of hydrate dissociation can be simply represented by the following kinetic model [45]:

(1) $$\frac{\partial m_{H}}{\partial t}=-kA_{S}(f_{eq}-f_{g})$$

where *m_H_* is the hydrate mass (kg); *A_S_* is the surface area of hydration reaction (m^2^); *k* is the constant of hydrate dissociation rate (kg/(m^2^·Pa·s)); and *f_eq_* and *f_g_* denote, respectively, the equilibrium fugacity and the actual fugacity of methane gas under the immediate temperature condition (Pa). The parameter *k* can be further calculated by *k* = *k*_0_exp(−Δ*E_a_*/*RT*), where *k*_0_ represents the intrinsic rate constant (kg/(m^2^·Pa·s)), Δ*E_a_* is the activation energy of hydrate dissociation (J/mol), *T* is the immediate temperature of gas hydrate (K), and *R* is the gas constant (J/(mol·K)). As the dissociation reaction occurs at the surface of the hydrate particles, the key parameter *A_S_* can be computed as

(2) $$A_{S}=F_{a}N_{V}(4\pi r_{p}^{2})S_{H}^{2/3}$$

where *F_a_* is the amendment factor of the surface area, *N_V_* is the number of voids of the hydrate-bearing sediments, *r_p_* is the radius of the sand particles, and *S_H_* is the hydrate saturation. The effectiveness of the kinetic model expressed in Equation (1) has been successfully verified by Li et al. [16], and, thus, it is also employed for simulating the kinetic dissociation of methane hydrate in the present study.

### 2.3. Domain Discretization

**Figure 2:**
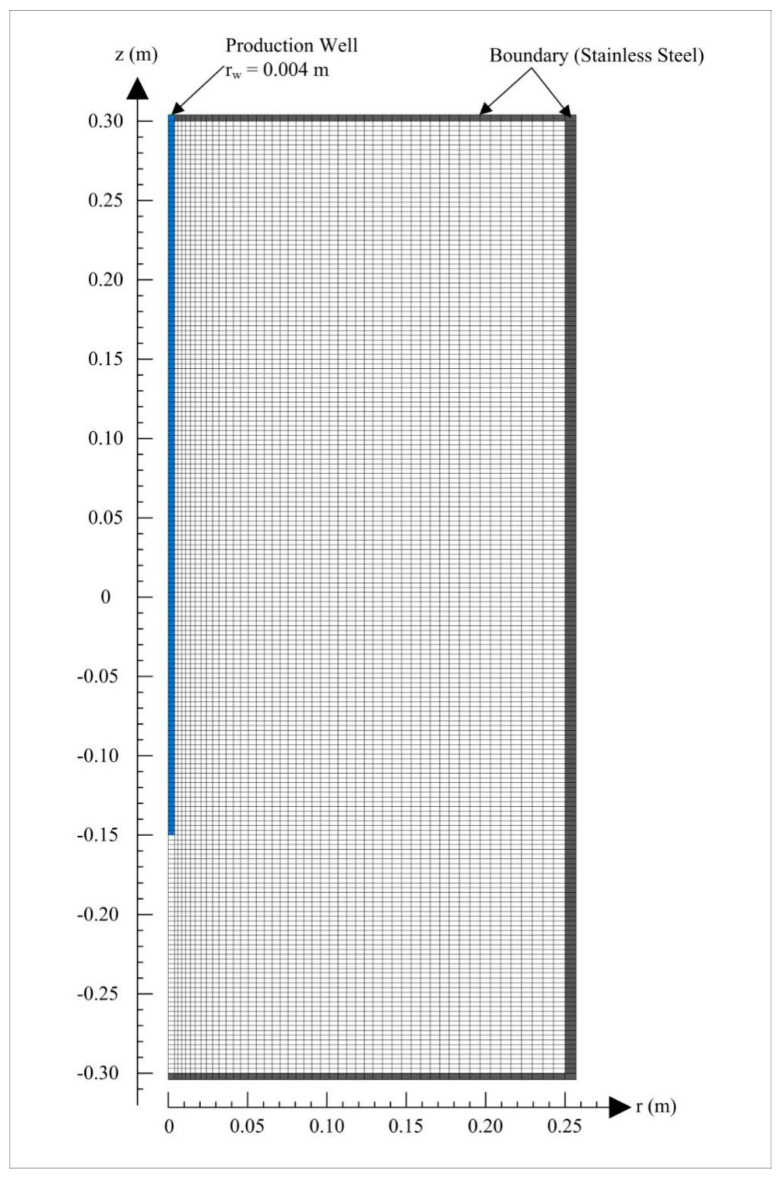
Two-dimensional cylindrical mesh for the numerical simulation.

Based on the axisymmetric property of the reactor, the PHS is discretized into a two-dimensional cylindrical mesh for the numerical simulation, and the detailed discretization pattern is shown in Figure 2. It is composed of 49 × 202 = 9898 elements in *r* and *z* directions in the cylindrical coordinate system. The number of elements in this mesh is about two times as large as that used by Li et al. [16], and it is aimed to investigate the dependence of the simulation results on the generated mesh. The stainless steel boundaries are represented by the outmost gridblocks (marked with black color in Figure 2), which are impermeable and inactive during the simulation. The wellbore section is represented by the elements located at the axis of the reactor within the region of −0.15 ≤ z ≤ 0.30 m (marked with blue color in Figure 2). Under the kinetic reaction mode without inhibitors, the above discretization will lead to a total of 39,592 coupled equations which must be calculated simultaneously to acquire the evolution of a series of primary variables in the whole domain.

### 2.4. Simulation Parameters for the PHS

The physical properties and simulation parameters of the hydrate reservoir are summarized in Table 2. The initial hydrate, gas and water saturations at the start of each simulation run are set with the value of *S_H_*_0_, *S_G_*_0_ and *S_W_*_0_, respectively, and they have been displayed in Table 1. The initial pressure and temperature of the inner elements in the PHS are set to be *P*_0_ and *T*_0_, respectively. To realize the constant depressurization conditions, the uppermost gridblock (*r* = 0 and *z* = 0.30 m) is set as a sink term by fixing its pressure at *P_Well_* during the whole simulation period. Other wellbore elements are handled as pseudo-porous media, of which the porosity is *ϕ_Well_* = 1.0, the intrinsic permeability is *K_Well_* = 5000 Darcies, and the capillary pressure is *P_cap_* = 0. No-flow conditions are assigned to the boundary elements by setting the permeability of them to be 0. The temperature of the boundary is initialized to be 7.00 °C, and it is fixed unaltered during the entire simulation period in each run. The porosity and intrinsic permeability of the sandy sediments are initialized to be *ϕ* = 0.435 and *K* = 50.0 Darcies, respectively. Other flow and wettability features of the hydrate deposit as well as the kinetic parameters presented in Table 2 are all originated from those used by Li et al. [16]. The duration of each simulation case is equivalent to the exploitation period of Δ*t* in Table 1.

## 3. Mathematical Model for Heat Transfer and Entropy Production

### 3.1. Calculation of Heat Transfer

The reaction of methane hydrate formation and decomposition is a reversible process, which is generally expressed as

(3) $${\mathrm{CH}}_{4}+N_{H}H_{2}O\Leftrightarrow {\mathrm{CH}}_{4}\cdot N_{H}H_{2}O\;+\;\Delta H_{m}$$

where *N_H_* is the hydration number, and Δ*H_m_* is the heat of hydration reaction for 1 mole methane hydrate (kJ/mol). Then the hydrate dissociation heat *Q_H_* is calculated as

(4) $$Q_{H}=\Delta H_{m}n_{H,diss}$$

Here, *n_H_*_,*diss*_ is the mole number of dissociated hydrate (mol). The dependence of Δ*H_m_* on the temperature *T* (K) can be simply expressed by [45]

(5) $$\Delta H_{m}=C_{1}+C_{2}T$$

When the temperature is within the range of 273.15 K ≤ *T* ≤ 298.15 K, the constants of *C*_1_ and *C*_2_ are 56.60 and 1.68 × 10^−2^, respectively. This indicates that Δ*H_m_* can be approximately taken as constant if the temperature change is small. Thus, the temperature of *T_eq_* shown in Table 1 is used to calculate Δ*H_m_* in Equation (5).

When the hydrate dissociation by depressurization is performed above the freezing point, *Q_H_* is only associated with two kinds of heat sources: the total amount of heat transferred from the boundaries *Q_B_* and the sensible heat increase in the hydrate deposit *Q_S_*. The latter item actually consists of two parts: the sensible heat variation of the remaining materials in the reservoir *Q_S_*_,*res*_, and the sensible heat increase in the produced water and gas *Q_S_*_,*pro*_. According to the rule of energy conservation, the mathematical relationships of the above heat items should be

(6) $$Q_{H}=Q_{B}-Q_{S}$$

(7) $$Q_{S}=Q_{S,res}+Q_{S,pro}$$

The *Q_B_* and *Q_S_* can be calculated by

(8) $$Q_{B}=\int_{0}^{t}q_{B}dt$$

(9) $$Q_{S,res}=\int_{0}^{t}\left({∭}_{V}c_{p}\rho \frac{\partial T}{\partial t}dV\right)dt$$

(10) $$Q_{S,pro}=\int_{0}^{t}(c_{W}{\dot{m}}_{PW}+c_{G}{\dot{m}}_{PG})\cdot (T_{Well}-T_{eq})dt$$

where *q_B_* stands for the total heat flux conducted through the boundary (W); *c_p_* and *ρ* are the specific heat capacity (kJ/(kg·K) and the density (kg/m^3^) of the hydrate-bearing sediments, respectively; ${\dot{m}}_{PW}$ and ${\dot{m}}_{PG}$ are the water and gas production rate (kg/s), respectively; and *T_Well_* is the temperature of the uppermost wellbore element which acts as the sink term (°C). *q_B_*, *c_p_* and *ρ* are further determined by

(11) $$q_{B}=\iint_{S_{B}}\left[-\lambda_{B}\left(\frac{\partial T}{\partial r}+\frac{\partial T}{\partial z}\right)\right]dS$$

(12) $$\rho =\rho_{s}(1-\phi )+\phi (\rho_{H}S_{H}+\rho_{W}S_{W}+\rho_{G}S_{G})$$

(13) $$c_{p}=\frac{1}{\rho }[c_{s}\rho_{s}(1-\phi )+\phi (c_{H}\rho_{H}S_{H}+c_{W}\rho_{W}S_{W}+c_{G}\rho_{G}S_{G})]$$

where *λ_B_* is the heat conductivity at the boundary site (W/(m·K)), *S_B_* denotes the surface area of all the boundaries, and the subscript *s* means the quartz sand.

### 3.2. Entropy Production Model

The whole system is composed of the hydrate deposit (including the quartz sand and the gas, water, and methane hydrate in the pores) and the outside environment (the constant-temperature cooling device). According to the material compositions of the hydrate deposit, the total entropy production of the whole system (Δ*S_total_*) should be composed of the entropy generation of the water (Δ*S_W_*), the gas (Δ*S_G_*), the methane hydrate (Δ*S_H_*), the quartz sand (Δ*S_sand_*), and the surrounding environment (Δ*S_env_*). In other words,

(14) $$\Delta S_{total}=\Delta S_{W}+\Delta S_{G}+\Delta S_{H}+\Delta S_{sand}+\Delta S_{env}$$

The entropy production of water can be calculated as

(15) $$\Delta S_{W}=\Delta S_{OW}+\Delta S_{DW}+\Delta S_{PW}$$

where Δ*S_OW_*, Δ*S_DW_* and Δ*S_PW_* are the entropy production of the initial residual water in the pores, the retained water from hydrate decomposition, and the produced water from the well, respectively. They are determined by

(16) $$\Delta S_{OW}=\underset{V}{∭}\phi c_{W}\rho_{W}S_{OW}\ln\frac{T}{T_{0}}dV$$

(17) $$\Delta S_{DW}=\underset{V}{∭}\phi c_{W}\rho_{W}S_{DW}\ln\frac{T}{T_{eq}}dV$$

(18) $$\Delta S_{PW}=\int_{0}^{t}c_{W}{\dot{m}}_{PW}\ln\frac{T_{Well}}{T_{eq}}dt$$

where *S_OW_* and *S_DW_* represent the saturations of the original residual water and the retained water from hydrate decomposition, respectively. 

With respect to the entropy production of methane gas (Δ*S_G_*), it consists of the entropy generation of the original free gas in the pores (Δ*S_OG_*), the retained gas from dissociated hydrate (Δ*S_DG_*), and the produced gas from the well (Δ*S_PG_*). The calculation equations of them are as follows:

(19) $$\Delta S_{G}=\Delta S_{OG}+\Delta S_{DG}+\Delta S_{PG}$$

(20) $$\Delta S_{OG}=\underset{V}{∭}\phi c_{G}\rho_{G}S_{OG}\ln\frac{T}{T_{0}}dV$$

(21) $$\Delta S_{DG}=\underset{V}{∭}\phi c_{G}\rho_{G}S_{DG}\ln\frac{T}{T_{eq}}dV$$

(22) $$\Delta S_{PG}=\int_{0}^{t}c_{G}{\dot{m}}_{PG}\ln\frac{T_{Well}}{T_{eq}}dt$$

where *S_OG_* and *S_DG_* are the saturations of the original free gas and the retained gas after hydrate decomposition, respectively.

Considering that the mass of water and gas obtained from the well is smaller than that caused by hydrate dissociation [16], it is assumed that the recovered mass is only originated from the dissociated hydrate in the above equations. In addition, the entropy production due to gas expansion is not taken into account by assuming that the produced gas is retained in a virtual element which has constant pressure (*P_Well_*) and variable volume conditions in the numerical simulation. Such treatment is based on the consideration that the expansion process of the recovered gas is not directly associated with the hydrate dissociation.

As the decomposition temperature of gas hydrate is *T_eq_*, the entropy production due to hydrate dissociation is calculated by

(23) $$\Delta S_{H}=\frac{Q_{H}}{T_{eq}}$$

The entropy generation of the quartz sand in the PHS can be determined by the following integral calculation:

(24) $$\Delta S_{sand}=\underset{V}{∭}(1-\phi )c_{s}\rho_{s}\ln\frac{T}{T_{0}}dV$$

The surrounding environment comprises the cooling device, of which the temperature is maintained stable at *T_B_* during hydrate decomposition. Thus, the entropy generation of the ambient environment could be obtained by

(25) $$\Delta S_{env}=-\frac{Q_{B}}{T_{B}}$$

Incorporating the above equations into the integral finite difference framework of the T + H code, the evolutions of various heat flows and entropy productions with time can be obtained during methane hydrate dissociation under depressurization. All the temperature variables in these entropy equations should be transformed into Kelvin temperature.

## 4. Results and Discussion

### 4.1. Profiles of Gas Recovery and Hydrate Decomposition

**Figure 3:**
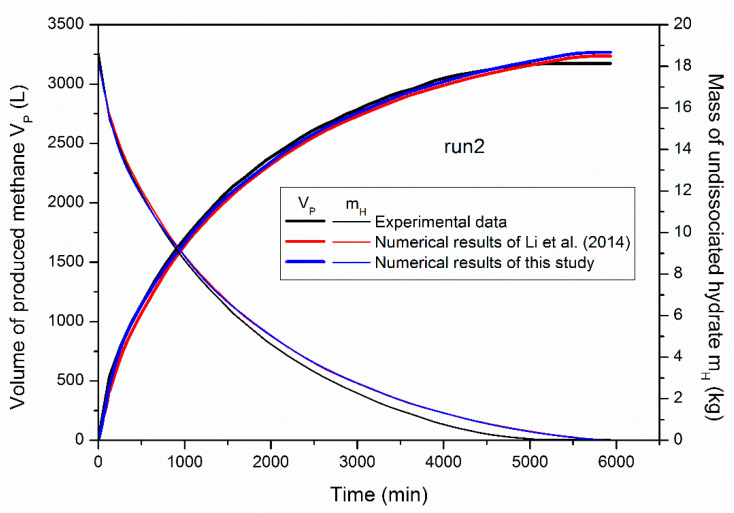
Comparison of the experimental data and the numerical results of *V_P_* and *m_H_* in run 2.

Figure 3 shows a comparison of the experimental data and numerical results of the cumulative volume of recovered methane (*V_P_*) and the residual mass of undissociated hydrate (*m_H_*) in run 2. Both the numerical results of Li et al. [16] and this study are displayed in this figure. One can notice that continuous gas production is obtained due to successive hydrate dissociation facilitated by the depressurization driving force, while the gas recovery rate declines with time owing to the weakened heat transfer rate from outside and the declined mass transfer rate from hydrate decomposition. Although the domain discretization in this work is finer than that of Li et al. [16], the simulated profiles of both *V_P_* and *m_H_* of the two studies present negligible differences, and they show good agreement with the experimental data. This further demonstrates the reliability of the employed code and the coupled heat transfer model for the description of the phase transition characteristics of methane hydrate in the PHS. Furthermore, the accuracy of numerical simulation is not strongly dependent on the discretization manner of the domain when the used mesh is refined enough. Therefore, the thermodynamic features including the distribution of temperature, pressure, and phase saturations in the PHS should also be identical to that obtained by Li et al. [16]. Readers who are interested in these results can be referred to this published literature. This work is primarily concentrated on the heat transfer and entropy production analyses as well as their intrinsic relationships with hydrate exploitation efficiency after the validation of the employed code.

### 4.2. Analysis of Heat Transfer Properties

**Figure 4:**
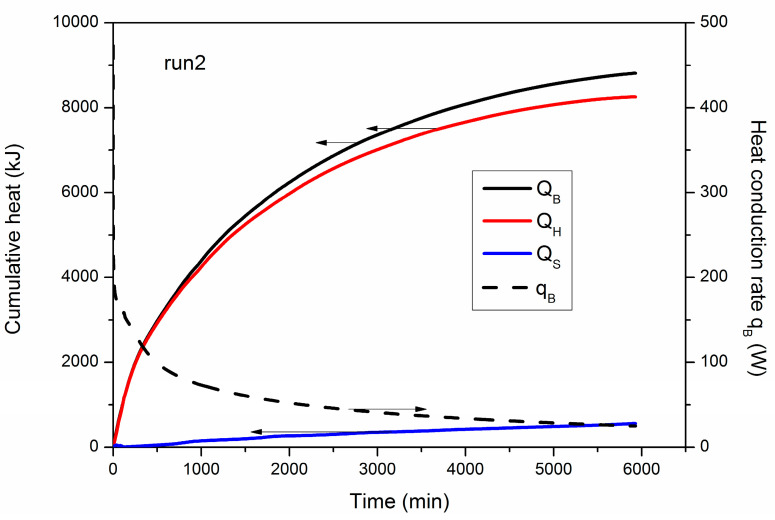
Evolution of *Q_B_*, *Q_H_*, *Q_S_* and *q_B_* during hydrate decomposition in run 2.

Figure 4 shows the numerical results of the evolution of *Q_B_*, *Q_H_*, *Q_S_* and *q_B_* during hydrate decomposition in run 2. The cumulative heat flows and entropy productions at the end of the three cases have also been summarized in Table 3. It is revealed from Figure 4 that *Q_H_* grows continually during the exploitation process, and it is only originated from the transferred heat from the outside environment [37]. When the heat is conducted into the hydrate deposit, most of *Q_B_* is absorbed and utilized by methane hydrate dissociation, while a small amount of heat (*Q_S_*) is consumed by the sandy sediments and the free gas and water stored in the porous media as well as the produced fluid from the well. On the other hand, the final *Q_S_* is 557.21 kJ at the end of run 2, and it only accounts for 6.32% of the final *Q_B_* (8813.55 kJ, Table 3). This indicates that the heat loss is relatively small in comparison to *Q_H_*. This is caused by the limited temperature difference between *T_B_* and *T*_0_ shown in Table 1. As the hydrate dissociation front gradually shrinks from the boundary towards to inner zones of the PHS [16], the resistance of heat transfer rises with time, which results in a fast decrease in *q_B_*, as shown in Figure 4.

**Figure 5:**
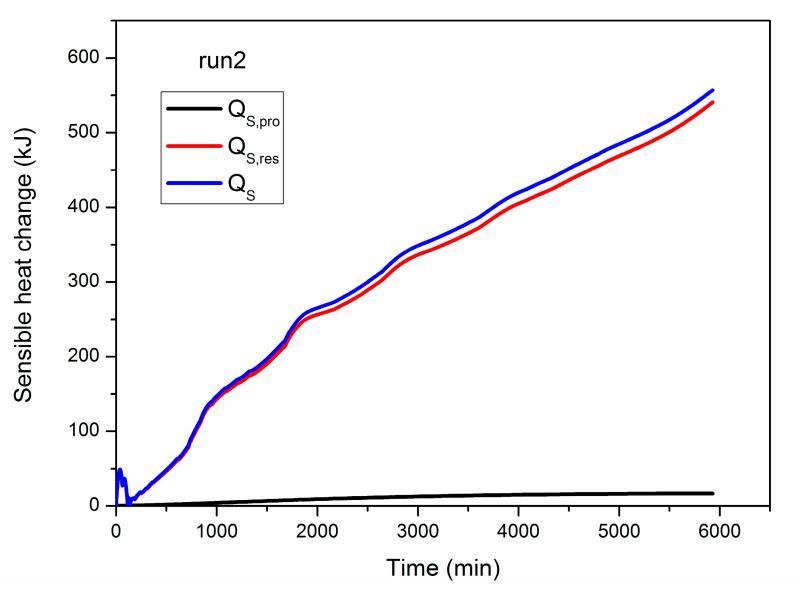
Numerical results of the sensible heat change of the produced mass (*Q_S_*_,*pro*_), the hydrate reservoir (*Q_S_*_,*res*_), and the whole system(*Q_S_*) in run 2.

As depicted in Equation (7), the sensible heat change of the entire system is composed of *Q_S_*_,*pro*_ and *Q_S_*_,*res*_, and the numerical results of them are given in Figure 5. It can be observed that the growth rate of *Q_S_*_,*res*_ is much higher than that of *Q_S_*_,*pro*_, which implies that the majority of the lost heat is captured by the hydrate reservoir and then stored as its extra sensible heat. This is caused by the fact that the mass of the produced fluid (methane gas and water) is much smaller than that of the entire hydrate deposit. Thus, the curve of *Q_S_*_,*res*_ shows ignorable difference with that of *Q_S_* in Figure 5. At the end of run 2, the final *Q_S_*_,*pro*_ is about 16.41 kJ, which only accounts for 2.95% of *Q_S_*. In other words, the heat loss caused by the fluid production is insignificant when compared with the heat absorption of the hydrate reservoir. The fluctuation observed in the early period in Figure 5 may be caused by the unstable hydrate decomposition when the blockage effect of solid hydrates on gas and water flow is more pronounced under higher hydrate saturation conditions.

For the purpose of characterizing the heat absorption performance of methane hydrate under depressurization, the heat utilization efficiency *ξ* is introduced as a relative criterion of the production efficiency. It is computed as the ratio of *Q_H_* to *Q_B_* by

(26) $$\xi =\frac{Q_{H}}{Q_{B}}$$

**Figure 6:**
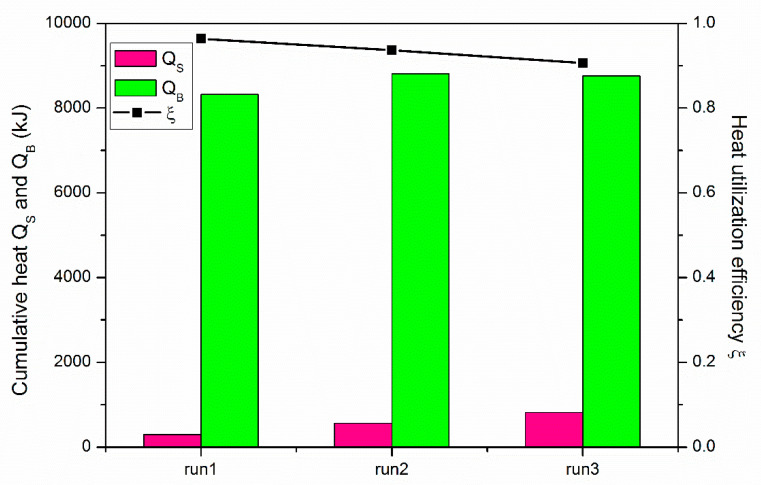
Comparison of *Q_S_*, *Q_B_* and *ξ* at the end of the three depressurization cases.

Figure 6 shows the comparison of *Q_S_*, *Q_B_* and *ξ* at the end of the three depressurization cases. The corresponding values of them have also been displayed in Table 3. One can see that *Q_S_* exhibits an increasing tendency as the wellbore pressure is dropped from 4.70 to 3.70 MPa in the three runs. This is because a lower *P_Well_* is accompanied by a lower initial temperature of the reservoir *T*_0_ (see Table 1), which leads to a faster heat transfer rate across the boundary and larger amount of heat loss to the residual materials in the vessel. Then the *Q_B_* also tends to rise slightly to supplement this part of additionally consumed heat by the hydrate deposit. On the other hand, as *Q_S_* is much smaller than *Q_B_* in each case, favorable heat utilization efficiency (more than 90.00%) has been obtained, which indicates that depressurization is an energy-efficient strategy of hydrate exploitation [29]. Because of the raised heat assimilation of the hydrate deposit, *ξ* shows a declined trend with the reduction of production pressure, as shown in Figure 6 and Table 3.

### 4.3. Analysis of Entropy Production Behaviors

**Figure 7:**
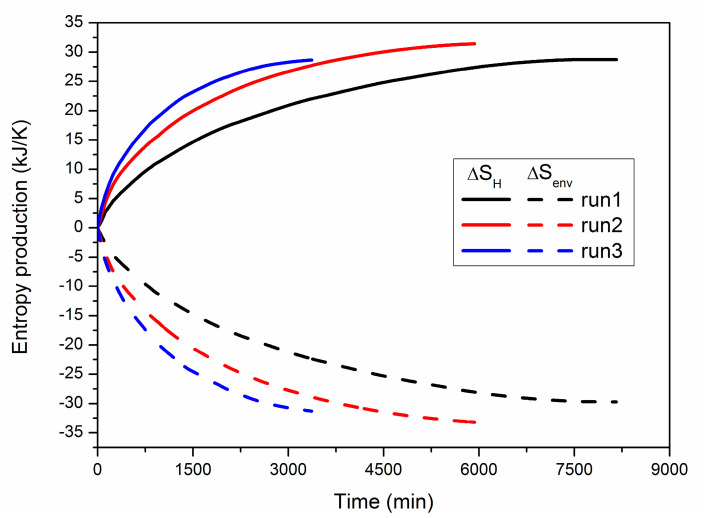
Numerical results of ∆*S_H_* and ∆*S_env_* during hydrate dissociation in the three cases.

Figure 7 shows the numerical results of ∆*S_H_* and ∆*S_env_* during hydrate exploitation under varying wellbore pressure in the three cases. The calculation methods of them are shown in Equations (23) and (25), respectively. As the hydrate dissociation is considered to occur at *T_eq_* in Table 1, and the temperature of the boundary *T_B_* is also maintained constant, the entropy productions ∆*S_H_* and ∆*S_env_* in each case are mainly controlled by the heat flows of *Q_H_* and *Q_B_*, respectively. As a consequence, the evolution trend of ∆*S_H_* seems to be very alike to that of *Q_H_* shown in Figure 4, while ∆*S_env_* is always negative because heat is continuously lost from the surrounding environment. The temperature deviation between the ambient environment and the inner reactor will become more significant under lower *P_Well_* conditions, which further causes faster increases in *Q_H_* and *Q_B_*. As a result, the increasing rate of ∆*S_H_* and the declining speed of ∆*S_env_* are both raised more remarkably under the conditions of lower wellbore pressure.

Because of the reversibility of the hydration reaction process shown in Equation (3), the assimilated heat *Q_H_* can be released again when the same amount of solid hydrate is formed at *T_eq_* in the PHS. Thus, ∆*S_H_* can be considered as the entropy flow from the ambient environment to the hydrate particles. However, as the heat transfer is a non-isothermal and irreversible process in the vessel, part of the transferred heat will be lost to gas, water and quartz sand, which results in unfavorable entropy production of these materials. Due to the similar *Q_H_* at the end of the three runs (Table 3), the final ∆*S_H_* in each case is also located at the same level, as shown in Figure 7. The absolute value of ∆*S_env_* is always larger than that of ∆*S_H_*, which is due to the heat consumption of the hydrate deposit.

**Figure 8:**
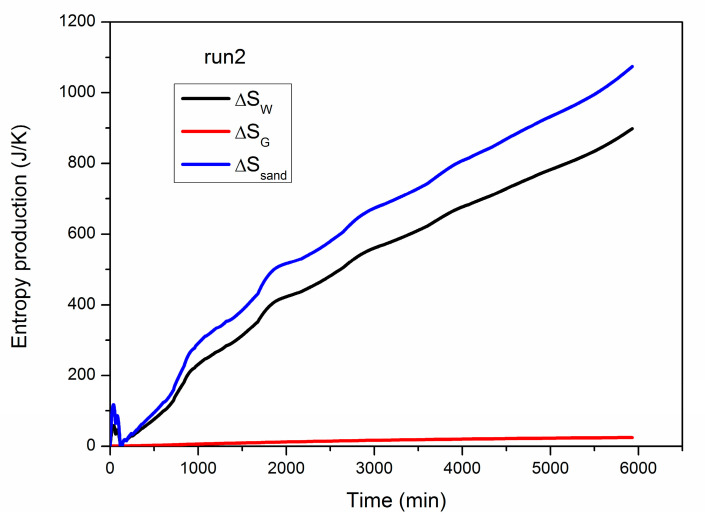
Evolution of ∆*S_W_*, ∆*S_G_* and ∆*S_sand_* during hydrate dissociation in run 2.

Figure 8 gives the evolution of ∆*S_W_*, ∆*S_G_* and ∆*S_sand_* during hydrate exploitation in run 2, and the final values of them are displayed in Table 3. As described in Figure 5, part of the heat transferred from the boundary is retained by the water, gas and quartz sand as sensible heat in the PHS. Thus, the curves of ∆*S_W_*, ∆*S_G_* and ∆*S_sand_* all increase persistently during the whole exploitation period. These entropy generations are caused by the irreversible heat conduction and thermal convection in the hydrate reservoir under notable temperature gradient. Comparatively, the rise of ∆*S_G_* is the least significant due to the smallest mass faction of methane gas in the system (the total amount of gas, water and quartz sand are about 3.35, 42.02 and 173.05 kg, respectively [16]), while ∆*S_sand_* rises with the fastest rate when the majority of the lost heat is captured and stored in the sand. Moreover, the final ∆*S_W_*, ∆*S_G_* and ∆*S_sand_* in run 2 are 897.98, 24.54 and 1073.80 J/K (Table 3), respectively, which are much lower than ∆*S_H_* of 31,417.80 J/K. Hence, the reversible heat flow of *Q_H_* is the major source for the entropy production of methane hydrate, and the entropy generation due to the irreversible heat flow is comparatively restricted.

**Figure 9:**
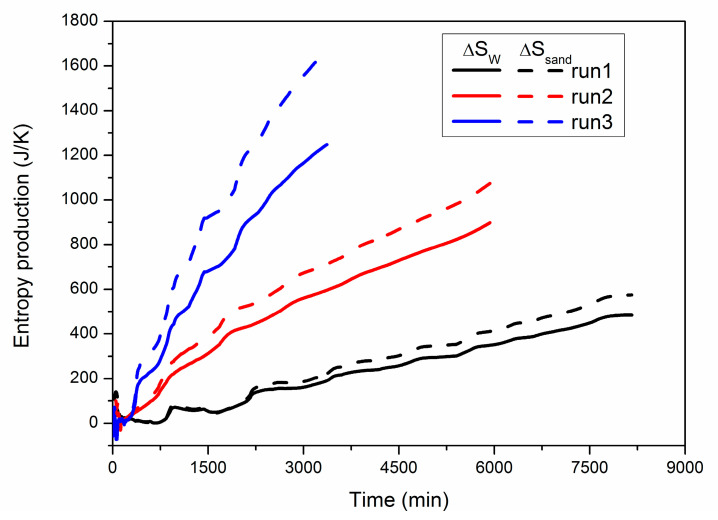
Numerical results of ∆*S_W_* and ∆*S_sand_* during hydrate dissociation in the three cases.

As ∆*S_W_* and ∆*S_sand_* are much higher than ∆*S_G_*, Figure 9 further shows the comparison of the evolution of the two entropy generations during hydrate dissociation under different production pressures. Similar with run 2, both ∆*S_W_* and ∆*S_sand_* show a continuous increase during the entire production process, while the rising rates of them are both enhanced to a higher level under lower production pressure due to faster heat consumption rate of the hydrate deposit, as discussed in Figure 6. In addition, the final difference between ∆*S_W_* and ∆*S_sand_* is only 89.67 J/K at *P_Well_* = 4.70 MPa, while it is raised to 175.82 J/K and 419.29 J/K at *P_Well_* = 4.20 MPa and 3.70 MPa, respectively, as presented in Table 3. This implies that the increase in ∆*S_sand_* is more significant at lower *P_Well_*, which is also caused by the more powerful thermal absorptivity of the hydrate-bearing sediments. Therefore, the energy loss can be mainly attributed to the irreversible heat conduction from the boundary to the quartz sand during hydrate dissociation under depressurization.

**Figure 10:**
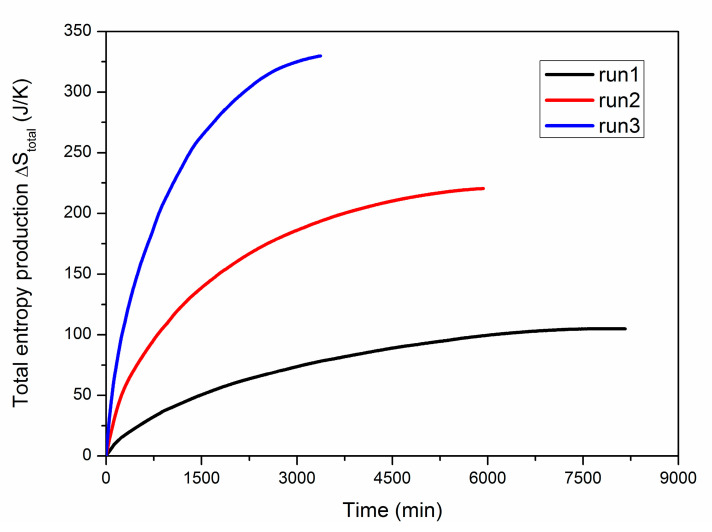
Profiles of the total entropy production ∆*S_total_* during hydrate dissociation in the three cases.

Figure 10 shows the profiles of the total entropy production ∆*S_total_* during gas recovery in the three runs. It is the sum of the five entropy productions presented in Equation (14). As expected, ∆*S_total_* is always larger than 0 due to the irreversibility of the non-isothermal heat transfer process in the reservoir. Although ∆*S_env_* is negative and its absolute value is the largest among the five items of entropy production (see Figure 7 and Table 3), the majority of it has migrated to the hydrate particles and then exists as ∆*S_H_*, which is favorable from the perspective of efficient hydrate exploitation. Thus, the total entropy production is mainly originated from the irreversible heat flows of *Q_S_*. It is revealed from Figure 10 that the entropy generation rate gradually decreases with time. This is caused by the reduced heat transfer rate in the PHS. In general, a lower wellbore pressure will lead to a larger ∆*S_total_*, which means higher irreversible energy loss of the whole system. Nevertheless, the ratios of ∆*S_total_* to ∆*S_H_* for runs 1–3 are only 0.36%, 0.70% and 1.15%, respectively. This indicates that the irreversible energy loss is extremely limited in comparison to the total quantity of energy assimilated by hydrate dissociation. This is in accordance with the results discussed in Figure 4 and Figure 6.

### 4.4. Analysis of Energy Recovery

The energy recovery rate *E_R_* is employed as another important criterion to evaluate the exploitation effect of the employed strategy. It is determined by

(27) $$E_{R}=\frac{H_{\mathrm{CH}4}}{t}$$

where *H*_CH4_ is the total combustion enthalpy of the recovered methane gas (J).

**Figure 11:**
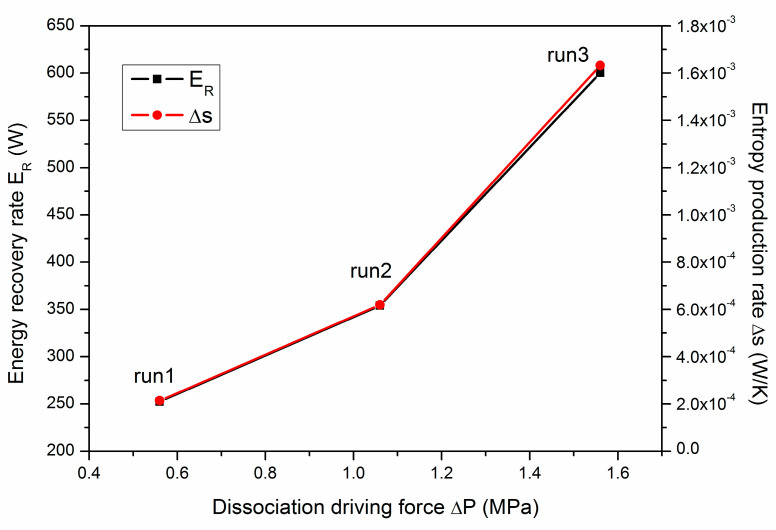
Dependence of the energy recovery rate (*E_R_*) and the entropy production rate (∆*s*) on the depressurization driving force (∆*P*).

Figure 11 presents the dependence of the final *E_R_* and the average entropy production rate (∆*s*) on the depressurization driving force (∆*P*). The depressurization driving force is defined to be ∆*P* = *P_eq_* − *P_Well_*, where *P_eq_* (about 5.26 MPa) is the equilibrium pressure at the temperature of *T_B_*. The corresponding values of ∆*P*, *E_R_* and ∆*s* can be also be found in Table 3. It is shown in Figure 11 that both *E_R_* and ∆*s* can be enhanced to a higher level under larger ∆*P*. This is because a lower production pressure is associated with a faster dissociation rate of gas hydrate, and the heat absorption rate of the deposit is also increased, as mentioned in Figure 6. In addition, the increasing amplitude of *E_R_* and ∆*s* is more remarkable under lower *P_Well_*. Although a higher entropy production rate of the whole system means faster irreversible energy loss in the depressurization process, the value of ∆*s* is about two orders of magnitude lower than that of the entropy production rate of methane hydrate. Thus, the lost energy is very limited, and a lower production pressure would be more beneficial for commercial hydrate development from the point of view of a faster energy recovery rate from hydrate deposits.

**Figure 12:**
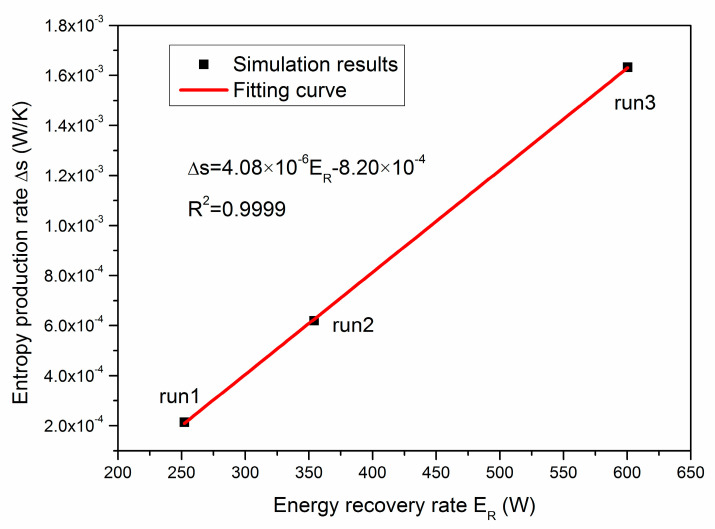
Relationship between the energy recovery rate (*E_R_*) and the entropy production rate (∆*s*).

The interactions between the energy recovery rate (*E_R_*) and the entropy production rate (∆*s*) at the end of the three runs are shown in Figure 12. It can be observed that ∆*s* rises with the increase in *E_R_*. As an approximate correlation, the relationship between *E_R_* and ∆*s* can be fitted with the following linear function:

(28) $$\Delta s=4.08\times 10^{-6}E_{R}-8.20\times 10^{-4}\begin{array}{cc} (W/K), & R^{2}=0.9999 \end{array}$$

The slope of the above linear equation is so slow that even a tremendous increase in *E_R_* would not cause sharp growth of ∆*s*. This further proves that the production pressure should be set as low as possible for the purpose of acquiring desirable *E_R_* while the irreversible energy loss is always controllable. This is in accordance with the result of Li et al. [47], who have found that the total energy investment would decrease with the rise of the depressurization driving force. It should be noted that such a conclusion could be only applied in situations when the wellbore pressure is set above the quadruple point (2.63 MPa, below which ice phase will be involved). This is because the heat transfer and entropy production models proposed in this study have not yet been taken into account the existence of ice in hydrate deposits. Future work shall be focused on the development of a more universal entropy production model for the thermodynamic analysis of more complex production scenarios with other advanced exploitation strategies.

## 5. Conclusions

The heat transfer and entropy production properties of methane hydrate dissociation by depressurization have been investigated in the PHS through numerical simulation. A mathematical model for the calculation of heat transfer and entropy generation is established, and it is incorporated into the T + H code to obtain the change behaviors of various heat flows and entropy productions under different production pressures.

The simulated results of both *V_P_* and *m_H_* show fine agreement with the experimental data and the numerical results reported in the literature, and the accuracy of numerical simulation is not strongly dependent on the discretization pattern of the domain when the used mesh is refined enough. The employed code and the coupled heat transfer model are certified to be capable of accurately describing the kinetic decomposition behaviors of methane hydrate in the PHS.

The heat consumption of gas hydrate *Q_H_* comes from the transferred heat from the ambient environment *Q_B_* during the stable depressurization stage. Most of *Q_B_* is absorbed and utilized by methane hydrate, while a small amount of heat is wasted as *Q_S_* by other materials in the system. The majority of the lost heat is captured by the hydrate reservoir and then stored as its extra sensible heat, while the heat loss caused by the fluid production is insignificant. Thus, *Q_S_* is much smaller than *Q_B_* in each case, and favorable heat utilization efficiency (more than 90.00%) has been obtained.

The total entropy production Δ*S_total_* should be composed of Δ*S_W_*, Δ*S_G_*, Δ*S_H_*, Δ*S_sand_*, and Δ*S_env_*. The entropy productions of ∆*S_H_* and ∆*S_env_* in each case are mainly dominated by the heat flows of *Q_H_* and *Q_B_*, respectively. ∆*S_H_* can be considered as the entropy flow from the ambient environment to the hydrate particles, and it is favorable from the perspective of efficient hydrate exploitation. On the contrary, ∆*S_W_*, ∆*S_G_* and ∆*S_sand_* are caused by the irreversible heat conduction and thermal convection under notable temperature gradient. Thus, the total entropy production is mainly originated from the irreversible heat flows of *Q_S_*. Compared with Δ*S_H_*, Δ*S_total_* is so low that the entropy generation, due to the irreversible heat flow, is largely restricted. This indicates that the irreversible energy loss is insignificant when gas hydrate is dissociated under depressurization.

Both the energy recovery rate *E_R_* and the average entropy generation rate ∆*s* will be raised to a higher level under larger depressurization driving force. However, the lost energy is very limited as the value of ∆*s* is about two orders of magnitude lower than that of the entropy production rate of methane hydrate. Therefore, the production pressure could be adjusted as low as possible to acquire desirable *E_R_* while the irreversible energy loss is always controllable.

## Figures and Tables

**Table 1:** Summary of experimental results of methane hydrate dissociation under stable depressurized conditions in the PHS [16].

Run	Δ*t* (min)	*P*_0_ (MPa)	*T*_0_ (°C)	*P_W__ell_* (MPa)	*T_eq_* (°C)	*S_H_*_0_	*S_G_*_0_	*S_W_*_0_	*T_B_* (°C)	*V_P_* (L)
1	8160	4.74	6.07	4.70	5.87	0.386	0.214	0.400	7.00	3113.7
2	5928	4.25	5.18	4.20	4.74	0.391	0.208	0.401	7.00	3174.4
3	3366	3.73	4.35	3.70	3.48	0.378	0.239	0.383	7.00	3053.8

**Table 2:** Physical properties and simulation parameters of the hydrate reservoir in the reactor.

Parameter	Value
Diameter of the simulator	0.50 m
Height of the simulator	0.60 m
Effective volume of the simulator	117.8 L
Porosity of the deposit *ϕ*	0.435
Boundary temperature *T_B_*	7.00 °C
Intrinsic permeability *K*	50.0 Darcies
Salinity	0
Intrinsic rate constant *k*_0_	4578 kg/(m^2^·Pa·s)
Hydration activation energy Δ*E_a_*	8.1 × 10^4^ J/mol
Area amendment factor *F_a_*	2.0
Wet thermal conductivity *k_ΘRW_*	3.1 W/(m·K)
Dry thermal conductivity *k_ΘRD_*	1.0 W/(m·K)
Composite thermal conductivity model [45]	*k_ΘC_* = *k_ΘRD_* + (*S_A_*^1/2^ + *S_H_*^1/2^)(*k_ΘRW_* − *k_ΘRD_*) + *ϕS_I_k_ΘI_*
Capillary pressure model [46]	*P_cap_* = −*P*_01_ [(S*)^−1/*λ*^ − 1] ^1−*λ*^
	S* = (*S_A_* − *S_irA_*)/(*S_mxA_* − *S_irA_*)
*S_irA_*	0.04
*λ*	0.45
*P*_01_	10^5^ Pa
Relative permeability model [45]	*k_rA_* = (S_A_^*^)*^n^*
	*k_rG_* = (S_G_^*^)*^nG^*
	S_A_^*^ = (*S_A_* − *S_irA_*)/(1 − *S_irA_*)
	S_G_^*^ = (*S_G_* − *S_irG_*)/(1 − *S_irA_*)
*n*	3.572
*n*_G_	3.572
*S_irG_*	0.287
*S_irA_*	0.200

**Table 3:** Numerical simulation results of heat transfer and entropy production in the three cases.

Run	∆*P*	*E_R_* (W)	*Q_H_* (kJ)	*Q_S_* (kJ)	*Q_B_* (kJ)	*ξ*	∆*S_W_* (J/K)	∆*S_G_* (J/K)	∆*S_H_* (J/K)	∆*S_sand_* (J/K)	∆*S_env_* (J/K)	∆*S_total_* (J/K)	∆*s* (10^−4^ W/K)
1	0.56	252.19	8024.67	299.58	8323.55	96.40%	484.65	12.17	28,739.60	574.32	−29,705.90	104.84	2.14
2	1.06	354.12	8256.34	557.21	8813.55	93.68%	897.98	24.54	31,417.80	1073.80	−33,193.70	220.42	6.20
3	1.56	600.35	7942.39	819.56	8761.95	90.65%	1248.17	27.53	28,662.50	1667.46	−31,275.90	329.77	16.33

## Nomenclature

*A_S_*	reaction surface area (m^2^)
*c_p_*	specific heat capacity (kJ/(kg·K))
*E_R_*	energy recovery rate (W)
*f*	fugacity (Pa)
*F_a_*	area amendment factor
*k*	dissociation rate constant (kg/(m^2^·Pa·s))
*K*	intrinsic permeability (m^2^)
*k_Θ__C_*	thermal conductivity (W/(m·K))
*k_ΘRD_*	thermal conductivity of dry porous medium (W/(m·K))
*k_ΘR__W_*	thermal conductivity of fully saturated porous medium (W/(m·K))
*k_ΘI_*	thermal conductivity of ice (W/(m·K))
*m*	mass (kg)
*n_H,diss_*	mole number of dissociated hydrate (mol)
*P*	pressure (MPa)
*P_Well_*	wellbore pressure (MPa)
*q_B_*	heat flow rate across the boundary (W)
*Q_B_*	total amount of heat transferred from the boundary (kJ)
*Q_H_*	total amount of hydrate dissociation heat (kJ)
*Q_S_*	sensible heat increase (kJ)
*Q_S,pro_*	sensible heat change of the produced fluid (kJ)
*Q_S,res_*	sensible heat change of the reservoir (kJ)
*r, z*	cylindrical coordinates (m)
*S*	phase saturation
*t*	time (min)
*T*	temperature (°C)
*V_P_*	volume of the recovered methane (L)
Δ*E_a_*	activation energy (J/mol)
Δ*H_m_*	hydrate dissociation heat (kJ/mol)
Δ*s*	entropy production rate (W/K)
Δ*S*	entropy production (J/K)
Δ*S_env_*	entropy production of ambient environment (J/K)
Δ*S_sand_*	entropy production of quartz sand (J/K)
Δ*S_total_*	total entropy production (J/K)
Δ*t*	duration of exploitation (min)
*ρ*	density (kg/m^3^)
*ϕ*	porosity
*ξ*	heat utilization efficiency
*Subscripts and Superscripts*	
0	represents initial condition
*B*	boundary
*cap*	capillary
*eq*	equilibrium
*G*	gas phase
*H*	solid hydrate phase
*irA*	irreducible aqueous phase
*irG*	irreducible gas
*n*	permeability reduction exponent - Table 2
*n_G_*	gas permeability reduction exponent - Table 2
*s*	sand
*W*	aqueous water phase
